# Five-Minute Awake Snoring Test for Determining CPAP Pressures (Five-Minute CPAP Test): A Pilot Study

**DOI:** 10.1155/2016/7380874

**Published:** 2016-01-11

**Authors:** Macario Camacho, Chad M. Ruoff, Makoto Kawai, Rahul Modi, Jabri Arbee, Anahid Hekmat, Matthew Robertson, Soroush Zaghi, Victor Certal, Robson Capasso, Clete A. Kushida

**Affiliations:** ^1^Department of Psychiatry and Behavioral Sciences, Division of Sleep Medicine, Stanford Hospital and Clinics, Redwood City, CA 94063, USA; ^2^Department of Otolaryngology, Division of Sleep Surgery and Medicine, 1 Jarrett White Road, Tripler Army Medical Center, Honolulu, HI 96859, USA; ^3^Department of Psychiatry and Behavioral Sciences, Stanford University, School of Medicine, Stanford, CA 94304, USA; ^4^Department of Otolaryngology-Head and Neck Surgery, Bharati Vidyapeeth Deemed University Medical College, Pune 411030, India; ^5^Department of Otolaryngology-Head and Neck Surgery, Division of Sleep Surgery, Stanford Hospital and Clinics, Stanford, CA 94304, USA; ^6^Department of Otorhinolaryngology, Sleep Medicine Centre, Hospital CUF, 4100-180 Porto, Portugal; ^7^Centre for Research in Health Technologies and Information Systems (CINTESIS), University of Porto, 4200-450 Porto, Portugal

## Abstract

*Objective*. To develop a quick, simple, bedside test for determining continuous positive airway pressures (CPAP) for obstructive sleep apnea (OSA) patients.* Study Design*. Prospective case series at a tertiary medical center.* Methods*. The Five-Minute Awake Snoring Test for Determining CPAP (Five-Minute CPAP Test) was developed and tested. Patients wear a soft-gel nasal triangle mask while holding a tongue depressor with the wide section (1.75 cm) between the teeth. Fixed pressure nasal CPAP is applied while the patient simulates snoring at 4 centimeters of water pressure. The pressure is incrementally titrated up and then down to determine the lowest pressure at which the patient cannot snore (Quiet Pressure).* Results*. Overall, thirty-eight patients participated. All could simulate snoring. Correlation coefficients were statistically significant between Quiet Pressures and body mass index (*r*_*s*_ = 0.60 [strong positive relationship], *p* = 0.0088), apnea-hypopnea index (*r*_*s*_ = 0.49 [moderate positive relationship], *p* = 0.039), lowest oxygen saturation (*r*_*s*_ = −0.47 [moderate negative relationship], *p* = 0.048), and oxygen desaturation index (*r*_*s*_ = 0.62 [strong positive relationship], *p* = 0.0057).* Conclusion*. This pilot study introduces a new concept, which is the final product of over one year of exploration, development, and testing. Five-Minute CPAP Test is a quick, inexpensive, and safe bedside test based on supine awake simulated snoring with nasal CPAP.

## 1. Introduction

Obstructive sleep apnea (OSA) is a common disorder with several medical treatment options (e.g., continuous positive airway pressure (CPAP) device use [1], myofunctional therapy [2], positional therapy, and weight loss) as well as several surgical treatment options (e.g., tonsillectomy [3], hypoglossal nerve stimulation [4], tracheostomy [5], and several other soft tissue [6] and bony surgeries [6]). Sullivan et al. published a landmark article in 1981 introducing CPAP as a new treatment for OSA [1]. Currently, a simple, bedside test for empirically prescribing CPAP devices as treatment for OSA patients is lacking. Techniques for prescribing CPAP have been studied to include overnight in-lab manual CPAP titration (CPAP titration) [7], autotitrating-CPAP (Auto-CPAP) [8], predictive mathematical equations [9], split-night sleep studies [10], and performing CPAP titration during a daytime nap [11]. With the rising costs of medical care, CPAP titration study reimbursement by third-party payers is becoming more difficult to obtain. The Agency for Healthcare Research and Quality (AHRQ) identified that developing more cost-effective strategies for managing CPAP is a high-priority for future OSA research projects [12]. For patients who are prescribed inadequate positive airway pressures (PAP), the cardiovascular complications and daytime sleepiness related to the OSA may persist. Therefore, additional studies exploring tests or techniques to improve success of CPAP titration and to empirically prescribe CPAP are needed.

A method for determining pressures for CPAP based on a quick test that can be performed on a patient during the office visit would be ideal. It is known that male sex, older age, increasing body mass index (BMI), nasal obstruction, micrognathia, hypertrophic palatine tonsils, macroglossia, supine body position, and other physiological characteristics can predispose patients to obstructive sleep apnea and can affect treatment outcomes [13]. BMI, neck circumference, apnea-hypopnea indices (AHI), cephalometrics, and other variables have been used in predictive positive airway pressure (PAP) mathematical equations [14–16]. One of the challenges of using predictive equations to prescribe CPAP is that the equations are generally specific to the population studied during the developmental phase [9]. Additionally, CPAP during sleep is intended to eliminate obstructive respiratory-related events (including snoring). If loud or unambiguous snoring is detected for ≥1 minute during a CPAP titration, then the recommendation is to increase the CPAP by ≥1 centimeter of water pressure (cwp) until the events are eliminated [7]. In clinical practice, the report of snoring from a bed partner while the CPAP device is being used is considered an indication that the prescribed pressure is too low. Therefore, one area of potential research is to incorporate snoring into a bedside test that can be used to determine CPAP treatment pressures.

Snoring has been defined as a respiratory sound that originates during sleep, which is caused by tissue vibrations and repeated airway collapse at the level of the pharyngeal airway [17, 18]. During sleep, spontaneous snoring has been noted with nasal and/or oral breathing [19]. Snoring is due to a combination of negative intra-airway pressures along with decreased pharyngeal muscle tone during inspiration (and sometimes during expiration) while the upper airway collapses passively [20]. During simulated snoring while the patient is awake, the size of the pharyngeal lumen decreases secondary to active muscle contraction of the palate and pharyngeal walls [20]. In OSA patients, the simple act of changing from the upright to the supine position causes posterior displacement of the tongue with an associated decrease in the airway cross-sectional area by 50% at the level of the retro-lingual airway and 32% at the level of the base of tongue [21]. Therefore, there is the potential to use simulated snoring in a bedside test.

The objective of this pilot study was to develop a safe, inexpensive, quick, and easily reproducible bedside test that can be performed in awake patients for determining pressures for CPAP in patients with OSA.

## 2. Materials and Methods

The Stanford University Institutional Review Board approved the protocol prior to the initiation of this prospective study. Inclusion criteria were as follows: (1) adults (≥18 years old) treated at the Stanford Sleep Medicine Clinic, (2) the patient was diagnosed with OSA, and (3) the clinic visit allowed sufficient time to inform, counsel, and consent the patient. Exclusion criteria were as follows: (1) the patient could not produce a simulated snoring sound (despite being taught) using maximal forced inspiratory effort [22] while sitting and then again in the supine position, (2) the patient had central sleep apnea, and (3) the patient had significant medical comorbidities such as a recent stroke or myocardial infarction. This study was conducted in three phases (Phase 1: development and testing, Phase 2: comparison of snoring during the simulated snoring testing to polysomnogram snoring, and Phase 3: prospective use of the test in patients being prescribed empiric CPAP).

In Phase 1, numerous maneuvers and techniques were developed and tested (including simulated snoring) that could be performed at the bedside during routine clinical practice. We also tested how the maneuvers, including simulated snoring, performed while fixed pressure CPAP was applied via different mask interfaces (nasal pillows, nasal triangles, and oral-nasal masks). After testing dozens of potential techniques, we determined that the supine awake simulated snoring test (which we have named as Five-Minute Awake Snoring Test for Determining CPAP Pressures [Five-Minute CPAP Test]) provided the best balance between simplicity, comfort, reproducibility, and potential clinical utility. After several attempts to standardize the patients with either a closed or an open mouth, it was determined that an open mouth was best as it allowed the patients' tongues to relax posteriorly and made it easier for snoring sound production. Therefore, we standardized the distance the mouth was held open by using a standard tongue depressor between their teeth. Because testing in Phase 1 concluded that the Five-Minute CPAP Test was the best new test overall, it was therefore used for Phases 2 and 3.

**Figure 1:**
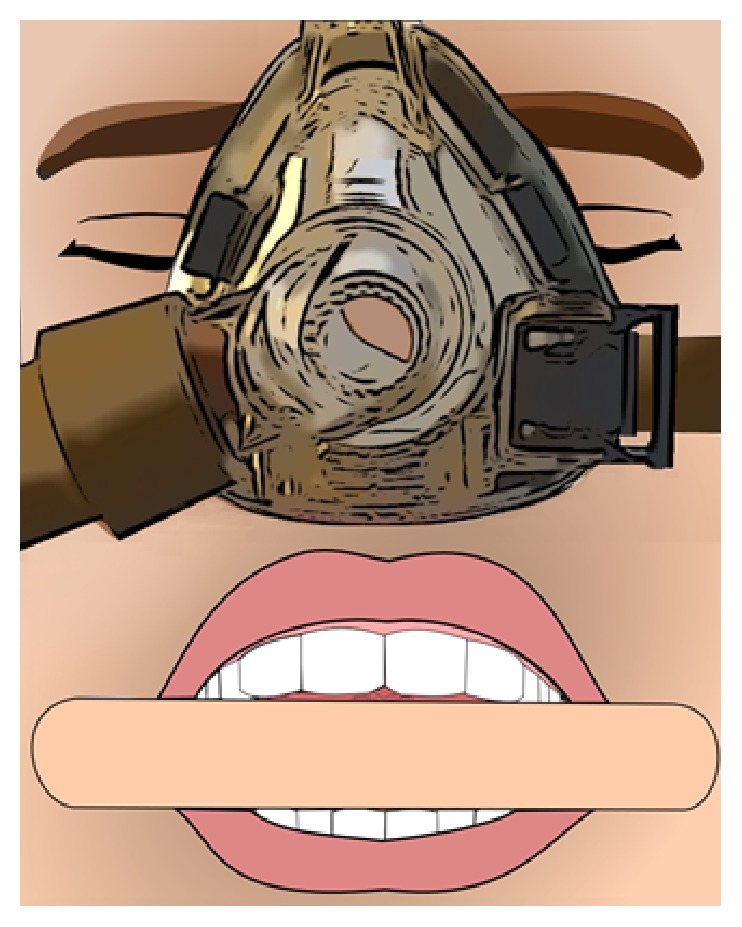
The Five-Minute CPAP Test setup. Front view of proper positioning of the soft-gel nasal triangle mask and tongue depressor (1.75 cm wide) which is placed between the teeth of the upper and lower jaw in order to standardize the distance the mouth is open.

**Figure 2:**
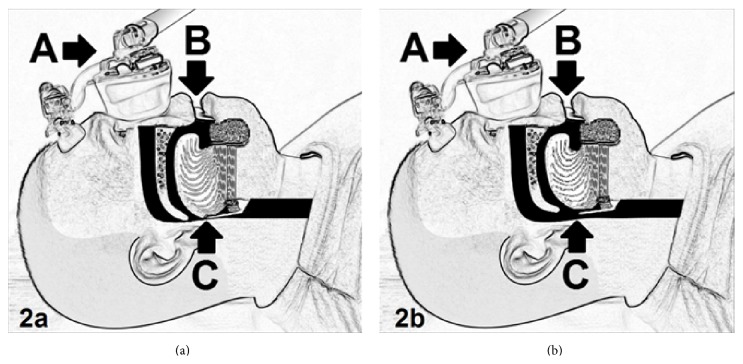
Fixed pressure delivered with a CPAP device (applied via a nasal soft-gel triangle mask) with the patient performing the Five-Minute CPAP Test using supine awake snoring. (a) demonstrates that the patient is able to snore at lower pressures with closure of the upper airway. (b) demonstrates a higher pressure at which the patient can no longer snore (Quiet Pressure) with an open airway. Labels: A = CPAP via a soft-gel nasal triangle mask, B = standard tongue depressor between teeth, and C = upper airway at the level of the soft palate, base of tongue, and epiglottis.

The Five-Minute CPAP Test is performed in several steps; see Figures 1 and 2 and Table 1 for the protocol used. First, the patient is asked to simulate snoring while sitting; then they are asked to lie supinely and simulate snoring again. Patients simulate snoring without the mask first and then again after the mask is applied. A CPAP device (in this study: a ResMed S9 [San Diego, CA, USA]) is set at the lowest fixed pressure of 4 cwp and the pressure is administered via a soft-gel nasal triangle mask. The mouth held open at a fixed distance (approximately 1.75 centimeters) using a standard tongue blade such that the wide part of the blade is held in position between the upper and lower teeth bilaterally.  Figure 1 demonstrates how the nasal-CPAP and tongue depressor are set up in a patient. The positive pressure is applied at 4 cwp. The patient (lying supinely) is asked to take three breaths and then simulate snoring. If the patient can still snore with positive pressure, the tongue depressor is removed from the patient's mouth and the pressure is increased by 2 cwp. The patient is allowed to swallow, then the tongue depressor is placed back into position, and the patient takes three breaths at the new pressure setting and simulates snoring again. This procedure is repeated until the pressure at which the patient cannot snore is defined. Once the pressure at which the patient cannot snore is defined from a starting pressure of 4 cwp and increasing in 2 cwp increments, the next step is to explore whether the patient can snore if the pressure is decreased by 1 cwp. If the patient can snore at the lower pressure, then the pressure is raised by 1 cwp to reconfirm that the patient cannot snore at this pressure. The Quiet Pressure is defined as the lowest pressure at which the patient cannot snore.  Figure 2(a) demonstrates a pressure that allows for snoring, and Figure 2(b) demonstrates a pressure that does not allow for snoring (Quiet Pressure).

In Phase 2, the Five-Minute CPAP Test was performed on patients presenting for CPAP titration studies. Testing was performed before the CPAP titration commenced, allowing for a comparison between the Quiet Pressure and variables from the polysomnograms (PSG). The American Academy of Sleep Medicine (AASM) Manual for the Scoring of Sleep and Associated Events [23] (2013, version 2.0.2) was used to analyze the PSG except hypopneas were scored if ≥30% reduction in airflow is measured by the nasal flow transducer lasting ≥10 seconds and associated with a 3% desaturation and/or an electroencephalogram arousal (standard for Stanford patients). The SleepSense (SLP Inc., Tel-Aviv, Israel) Piezo Snore Sensor was used for snoring detection in all of the PSG and the lowest pressure eliminating snoring was documented. The board-certified sleep physicians interpreting these in-laboratory CPAP titration were completely blinded from all details about this study, including the Quiet Pressures derived from this study.

In Phase 3, patients returning to the clinic to discuss their diagnostic sleep study results were enrolled for the Five-Minute CPAP Test if they were ineligible for a CPAP titration based on preauthorization denial by third-party payers or they specified that they wanted the same day empiric CPAP device prescription and they did not have central sleep apnea nor significant comorbidities. After reviewing the results of the sleep study with each patient, performing the Five-Minute CPAP Test to determine the Quiet Pressure, an Auto-CPAP prescription was generated at a variety of pressure ranges, which included the Quiet Pressure, to identify optimal pressure ranges above and below the Quiet Pressure. The patients were then seen after one month for a routine follow-up visit and to assess their Auto-CPAP device compliance. The patients' previously defined Quiet Pressures were compared to their demographic data, their diagnostic polysomnogram variables, and their Auto-CPAP devices' 95th percentile download data.

## 3. Statistics

The IBM Statistical Package for Social Sciences software (SPSS) version 22 (Armonk, New York, USA) was used for statistical analyses. Means and standard deviations (M ± SD) were calculated for demographic data, polysomnographic data, and Auto-CPAP device download data. Given that Spearman's rank correlation coefficient (*r*_*s*_) can be used for both discrete and continuous variables and is less sensitive to strong outliers (important for small sample sizes), it was selected for comparing the individual patients' Quiet Pressures to other study variables. The *r*_*s*_ strengths were categorized based on standard recommendations [24] of 0.0–0.19 = very weak, 0.20–0.39 = weak, 0.40–0.59 = moderate, 0.60–0.79 = strong, and 0.80–1.0 = very strong. A two-tailed *p* value <0.05 was considered statistically significant.

## 4. Results

Overall, thirty-eight patients participated in this pilot study (twenty-one patients in Phase 1, ten patients in Phase 2, and seven patients in Phase 3). All thirty-eight patients were able to simulate the snoring sound; therefore, no patient was excluded from this study. Phase 1 (development): after testing several oral-nasal, nasal triangle, and nasal pillow mask interfaces, the oral-nasal masks did not allow the patients to make clearly audible sounds through the masks and the soft-gel nasal triangle masks provided the best assessment of sound production by the patients when compared to other nasal triangle or nasal pillow masks; therefore the soft-gel nasal triangle masks were selected as the mask interface for this study. The Five-Minute CPAP Test was developed and progressively modified to improve it so that it was standardized, quick, and easy to perform. The average amount of time to perform the test was approximately ten minutes when initially learning to perform the technique and five minutes after performing the technique a few times, as described in Section 2. Phase 2: the Five-Minute CPAP Test was performed on ten patients presenting for a CPAP titration. The Quiet Pressures' M ± SD were 11.4 ± 2.0 cwp and snoring resolved during the CPAP titration studies' M ± SD were 12.8 ± 1.2 cwp, *r*_*s*_ = 0.37, and *p* value = 0.29.  Table 2 presents patient demographics, Quiet Pressures, CPAP titration snoring pressures, and the Auto-CPAP prescription pressures.

In Phase 3, a variety of pressure ranges surrounding the Quiet Pressure (e.g., ±2 cwp from the Quiet Pressure) were prescribed to determine the ideal Quiet Pressure Auto-CPAP prescription; see Table 3. The Quiet Pressures and the 95th percentile pressures were within 1-2 cwp of each other for all patients. The combined Quiet Pressures and the 95th percentile pressures were 10.9 ± 1.6 cwp and 10.7 ± 1.5 cwp, respectively (*r*_*s*_ = 0.51, *p* value = 0.19). For patient #7 in Table 3, who had the broadest range of pressures prescribed for Auto-CPAP (4–20 cwp), his Quiet Pressure and 95th percentile pressure were 10 cwp and 9.7 cwp, respectively.  Table 4 presents the overall combined data for the patients' Quiet Pressures as correlated with (1) demographic variables (age, BMI), (2) polysomnographic variables (AHI, LSAT, and oxygen desaturation index [ODI]), (3) the APAP devices' 95th percentile download data, (4) snoring detected on the polysomnograms, and (5) the CPAP titration prescription pressures (recommended low Auto-CPAP setting). Combining the data from the patients in Phases 1 and 2 demonstrates that the correlation coefficients were statistically significant between Quiet Pressures and BMI (*r*_*s*_ = 0.60 [strong positive relationship], *p* = 0.0088), AHI (*r*_*s*_ = 0.49 [moderate positive relationship], *p* = 0.039), LSAT (*r*_*s*_ = −0.47 [moderate negative relationship], *p* = 0.048), and ODI (*r*_*s*_ = 0.62 [strong positive relationship], *p* = 0.0057).

After testing multiple pressure combinations, the preliminary results from this pilot study suggest that the best balance between comfort and efficacy is the Quiet Pressure ±2 cwp, and this could potentially be used to prescribe empiric CPAP (see Table 5).

## 5. Discussion

There is a need to develop simple bedside techniques to better guide clinicians determining CPAP, such as when starting CPAP in titration studies or for prescribing CPAP empirically. It has been demonstrated that when starting CPAP titration, having a pressure that is closer to the therapeutic pressure from the beginning can improve the success rates for titration studies [9]. This pilot study demonstrates preliminary data for the Five-Minute CPAP Test, a bedside, awake simulated snoring test that uses fixed pressure CPAP and is a quick, inexpensive, simple, and safe technique to help define pressure ranges for CPAP. It requires only 5–10 minutes to perform, along with a tongue depressor, a nasal triangle mask, and a CPAP device. It does not require extensive training nor expertise in sleep medicine. The tongue depressors used are disposable, inexpensive (less than one to a few cents per tongue depressor), and easy to position, as described in Figure 1. We had no complications nor adverse events during this study. To our knowledge, this is the first bedside test that does not require any calculations and can be quickly performed in clinic. Given that the test utilizes the patients' own anatomy and physiology, we believe that the Five-Minute CPAP Test provides a reasonable method for helping to determine starting pressures for CPAP devices, such as at the beginning of CPAP titration studies, or for empiric CPAP prescriptions.

There are four main findings in this study. First, the Five-Minute CPAP Test can be easily performed in patients who are naïve to CPAP. In this study, all thirty-eight patients were able to simulate snoring. The use of nasal-CPAP with an open mouth as part of the Five-Minute CPAP Test allows for better assessment of sound production made by the patients. By keeping an open mouth while simulating snoring in the supine position, there is an increased ability of the tongue to fall posteriorly. By using a standard tongue depressor, we have standardized the distance the mouth is kept open, which improves reproducibility. Additionally, the Five-Minute CPAP Test provides patients the opportunity to experience CPAP prior to obtaining the device from the durable medical equipment (DME) company and provides office staff the opportunity to educate and answer patient questions prior to obtaining the CPAP device from the DME company.

Second, it is known that high pressures can make positive airway pressure devices more difficult to tolerate (e.g., high leak and/or aerophagia), while at the same time, patients who have very low pressures may still have flow limitation, snoring, hypopneas, apneas, oxygen desaturations, and limited subjective improvement. Therefore, a simple technique such as the Five-Minute CPAP Test to prescribe more therapeutic CPAP pressures from the onset could increase the time patients are in the therapeutic pressure range. As an example, patient #7 in Table 3, who had a Quiet Pressure of 10 cwp, had a 95th percentile pressure from the Auto-CPAP data download of 9.7 cwp during his clinic visit. If the patient had been initially prescribed Auto-CPAP using the Quiet Pressures ±2 cwp method, then his prescribed range of pressures would have been from 8 to 12 cwp, thereby enabling the device to operate at more therapeutic pressures (i.e., the 95th percentile) for more time.

Third, in the statistical analyses of patients' Five-Minute CPAP Test data, the correlation coefficients were statistically significant between Quiet Pressures and BMI (strong positive relationship), AHI (moderate positive relationship), LSAT (moderate negative relationship), and ODI (strong positive relationship). The combined Quiet Pressures and the 95th percentile pressures M ± SD were 10.9 ± 1.6 cwp and 10.7 ± 1.5 cwp, respectively (*r*_*s*_ = 0.51), moderately correlated, *p* value = 0.19 (although moderately correlated, likely statistical significance was not achieved secondary to a smaller sample size). In evaluating additional statistical testing, we considered utilizing the receiver operating characteristic (ROC) for Quiet Pressures and the CPAP titration polysomnogram (the “gold standard”); however, given the fact that this test is not a replacement for CPAP titration, we believe that comparing the two is inappropriate. Additionally, it would be difficult to compare a single number (Quiet Pressure) against an Auto-CPAP prescription which is a range of pressures. This test cannot and should not be thought of as a replacement for an overnight in-lab manual CPAP titration study [7] for determining optimal CPAP. Goals during this study were (1) to develop a safe test, (2) to avoid having to perform mathematical calculations as part of the prescription process, and (3) to make the test simple enough that it could easily be replicated. The results from this pilot study suggest that the Quiet Pressure could be incorporated into prescriptions, in a manner such as by adding and subtracting 2 cwp from the Quiet Pressure when prescribing Auto-CPAP.

Fourth, the goals during this study were (1) to avoid having to perform mathematical calculations as part of the prescription process, (2) to make the test simple enough that it could easily be replicated, and (3) to be able to write empiric CPAP prescriptions based solely on the results of the new bedside test. The test was designed to be performed in a standardized manner, with the tongue depressor holding the mouth open at a standardized distance for reproducibility. The Five-Minute CPAP Test was easily learned by physicians and sleep medicine technologists. Given that a range of pressures is prescribed, our goal would be that the Quiet Pressure is strategically placed between the low prescription pressure, allowing patients to better tolerate the device, and the high prescription pressure, allowing each device to effectively treat the OSA. After exploring several pressure ranges, we selected to have the prescription be the Quiet Pressure ±2 cwp to help naïve patients acclimate to the CPAP devices. In Table 5, the prescriptions shown for Auto-CPAP start at a low prescription range of 6–10 cwp and a high prescription range of 11–15 cwp. However, each institution can easily adjust their upper and lower limits.

There are limitations to this study. The sample size was limited; however, as a pilot study, our goal was to explore several potential techniques and once one technique was selected and fully developed, then it was important to demonstrate safety and the potential utility. In this study we introduce a new technique that involved multiple providers and over a year of field-testing. Second, it is known that head and neck physical exam findings such as nasal septal deviations, inferior turbinate sizes [25], Friedman palate position [26], tongue sizes, tonsil sizes, and nasal surgery [27] can contribute to prescription pressures and/or affect CPAP use. Although we did not evaluate these history and physical exam findings as part of this study, these variables could be investigated in future studies. Third, we did not explore whether the Quiet Pressure itself could be used for prescribing fixed-CPAP devices; this too could be explored in future research.

## 6. Conclusion

This pilot study introduces a new concept, which is the final product of over one year of exploration, development, and testing. The Five-Minute CPAP Test is a quick, inexpensive, and safe test based on supine awake simulated snoring with fixed nasal-CPAP. Additional research could help better determine the use in CPAP titration studies and/or for empiric CPAP prescriptions.

## Figures and Tables

**Table 1:** A protocol for performing the Five-Minute CPAP Test and determining the Quiet Pressure.

(1) The patient is asked to make a simulated snoring sound in the upright and then in the supine positions (if they cannot, then they are not good candidates for this test).	
(2) The nasal triangle soft-gel mask is applied.	
(3) A tongue blade is placed between the upper and lower teeth.	
(4) Fixed pressure CPAP is applied at 4 cm of water pressure (cwp).	
(5) The patient takes 3 breaths at 4 cwp and then simulates snoring.	
(6) If the patient can snore, then increase the pressure by 2 cwp.	
(7) The patient takes 3 breaths at the new setting and then simulates snoring.	
(8) If the patient can snore, then this process is repeated over and over by increasing (incrementally) the pressure by 2 cwp until they cannot simulate snoring (they are quiet).	
(9) The pressure is decreased by 1 cwp, the patient takes 3 breaths and then simulates snoring. If the patient can snore, then the pressure is increased by 1 cwp.	
(10) Once the lowest pressure at which the patient cannot snore is determined, this pressure is defined as the “Quiet Pressure.”	

**Table 2:** Quiet Pressures from the Five-Minute CPAP Test, snoring during titration study, and CPAP titration-based prescription (prescriber was blinded). % = percent; AHI = apnea-hypopnea index; BMI = body mass index (in kilograms per meter^2^); cwp = centimeters of water pressure; LSAT = lowest oxygen saturation; NA = not applicable (two patients started with CPAP and were later converted to bilevel, which they were prescribed); ODI = oxygen desaturation index; and y = years. Note: no patient had cardiac arrhythmias during the titration studies.

Patient	Age (y)/sex	BMI (kg/m^2^)	AHI (events/h)	ODI (events/h)	LSAT (%)	Five-Minute CPAP Test: Quiet Pressure (cwp)	Snoring resolved during titration (cwp)	CPAP Rx based on titration (cwp)
1	75/M	30.0	26.3	12.5	83	12	13	11–13
2	59/F	28.2	17.6	7.1	86	10	12	10–12
3	62/F	21.3	7.2	0.8	94	10	13	13–16
4	46/F	26.4	20.8	5.9	90	9	12	12–14
5	30/M	28.9	14.2	12.6	86	13	11	10–12
6	51/F	27.7	9.2	0.9	93	10	12	10–14
7	44/M	34.8	67.8	67.8	53	11	14	NA
8	21/M	31.6	43.8	21.9	86	16	15	NA
9	42/F	30.8	15.2	1.9	87	11	12	13–15
10	61/M	36.1	49	46.8	82	12	14	13–16
Total	49.1 ± 16.1	29.6 ± 4.2	27.1 ± 19.9	17.8 ± 22.4	84 ± 11.6	11.4 ± 2.0	12.8 ± 1.2	NA

**Table 3:** Data for patients who underwent the Five-Minute CPAP Test and empiric prescriptions, without a CPAP titration. % = percent; AHI = apnea-hypopnea index; BMI = body mass index (in kilograms per meter^2^); cwp = centimeters of water pressure; LSAT = lowest oxygen saturation; NA = not applicable; ODI = oxygen desaturation index; and y = years. ^*∗*^Based on previous prescription.

Patient	Age (y)/ sex	BMI (kg/m^2^)	AHI (events/h)	ODI (events/h)	LSAT (%)	Five-Minute CPAP Test: Quiet Pressure (cwp)	Auto CPAP: empiric Rx (cwp)	95th percentile pressures (cwp)
1	36/M	28.4	11.0	3.2	91	9	10–13	10.0
2	29/M	25.8	20.9	10.5	91	10	8–13	8.9
3	49/F	36.5	8.1	6.8	86	12	9–12	10.0
4	22/F	23.3	10.9	2.7	92	10	8–13	10.1
5	62/M	29.9	34.9	18.7	87	13	11–15	11.4
6	61/M	24.5	13.3	13.3	79	10	8–12	11.5
7	37/M	29.3	9.5	2.1	91	10	4–20	9.7
8	37/M	28	22.7	13.8	90	13	13–15^*∗*^	13.8
Total	41.6 ± 14.5	28.2 ± 4.1	15.5 ± 9.5	8.9 ± 6.1	88.4 ± 4.3	10.9 ± 1.6	NA	10.7 ± 1.5

**Table 4:** Spearman's rank correlation coefficient based on the Five-Minute CPAP Test Quiet Pressures versus other variables. ^*∗*^Statistical significance with 2-tailed *p* < 0.05. % = percent; AHI = apnea-hypopnea index; APAP-95th = Auto-CPAP devices' 95th percentile download data; BMI = body mass index (in kilograms per meter^2^); cwp = centimeters of water pressure; LSAT = lowest oxygen saturation; ODI = oxygen desaturation index; *p* = *p* value; *r*_*s*_ = Spearman's rank correlation coefficient; titration snoring = snoring detected during the polysomnogram; and y = years.

Quiet Pressure correlation to the following variables	*N* Correlation coefficient significance (2-tailed)
Age	*N* = 18 *r*_*s*_ = −0.0064 *p* = 0.98

BMI	*N* = 18 *r*_*s*_ = 0.60 *p* = 0.0088^*∗*^

AHI	*N* = 18 *r*_*s*_ = 0.49 *p* = 0.039^*∗*^

LSAT %	*N* = 18 *r*_*s*_ = −0.47 *p* = 0.048^*∗*^

ODI	*N* = 18 *r*_*s*_ = 0.62 *p* = 0.0057^*∗*^

APAP-95th	*N* = 8 *r*_*s*_ = 0.51 *p* = 0.19

Titration resolution of snoring in cwp	*N* = 10 *r*_*s*_ = 0.37 *p* = 0.29

**Table 5:** CPAP prescription based on the Five-Minute CPAP Test Quiet Pressures ±2 cm of water pressure (cwp). Note: at our institution we selected 7–11 cwp as the lowest prescription.

Quiet Pressure	CPAP prescription
≤8 cwp	6–10 cwp
9 cwp	7–11 cwp
10 cwp	8–12 cwp
11 cwp	9–13 cwp
12 cwp	10–14 cwp
≥13 cwp	11–15 cwp
